# Relationship between Helicobacter Pylori infection and metabolic syndrome components in adults

**DOI:** 10.3389/fendo.2025.1697797

**Published:** 2025-11-10

**Authors:** Yan-fei Jiang, Long Zhong, Jing-jing Guo, Qiao-fen Wang, Jia-xu Gu, Jia-yu Huang, Yan-jing Liu, Yuan Lin, Dong-hui Lu, Xiao-fen Lian

**Affiliations:** 1 Department of General Surgery, Shenzhen Luohu People’s Hospital, The Third Affiliated Hospital of Shenzhen University, Shenzhen, Guangdong, China; 2 Shenzhen University Medical School, Shenzhen University, Shenzhen, China; 3 Department of Urology, Peking University Shenzhen Hospital, Shenzhen, Guangdong, China; 4 Institute of Urology, Shenzhen Peking University-The Hong Kong University of Science and Technology Medical Center, Shenzhen, China; 5 Shenzhen Clinical Research Center for Urology and Nephrology, Shenzhen, China; 6 Department of Endocrinology, Peking University Shenzhen Hospital, Shenzhen, Guangdong, China; 7 Department of Rheumatology and Immunology, Hainan Affiliated Hospital of Hainan Medical University (Hainan General Hospital), Haikou, China; 8 Department of Dermatology, Peking University Shenzhen Hospital, Shenzhen, China; 9 Shantou University Medical College, Shantou University, Shantou, Guangdong, China

**Keywords:** metabolic syndrome component, Helicobacter pylori, inflammatory factors, HbA1c, diabetes, obesity

## Abstract

**Background and aim:**

Helicobacter pylori (H. pylori, HP) infection plays a significant role in the development and progression of various intra-gastric and extra-gastric diseases. Its infection is associated with numerous factors, including a series of metabolic-related diseases, and the potential connections between them remain highly controversial. Meanwhile, the prevalence of metabolic diseases has been increasing exponentially with changes in economic levels and lifestyles. Exploring the correlations and potential mechanisms between HP and metabolic diseases is crucial for future disease prevention and control. Due to the ongoing controversy surrounding its relevance and the absence of articles investigating the metabolic-related mediating mechanisms and threshold effects of related metabolic diseases leading to HP infection, this study holds significant importance for guiding future lifestyle and disease control.

**Methods:**

By collecting relevant test and examination indicators from 7,387 participants at Peking University Shenzhen Hospital, we analyzed the potential pathogenic mechanisms using statistical methods such as regression analysis, mediation analysis, and threshold analysis.

**Results:**

We found that factors such as blood glucose levels (fasting blood glucose (FBG), glycosylated hemoglobin (HbA1c)), Body Mass Index (BMI), and blood pressure (Systolic Blood Pressure (SBP), Diastolic Blood Pressure (DBP)) were the main risk factors influencing the target outcomes in this study, while higher levels of Albumin (Alb) may have a certain protective effect, with BMI playing a particularly significant role among these factors.

**Conclusion:**

This discovery has deepened our understanding of metabolic diseases, BMI, related metabolic indicators, and HP infection.

## 1 Introduction

H.pylori is one of the oldest and most prevalent members of the human gastric microbiota, and has co-evolved with humans and persistently colonizes the stomach. It affects over half of the global population, with its prevalence showing an upward trend among adolescents and children, which is particularly concerning (1). Beyond its well-known impact on gastrointestinal diseases, such as gastric cancer, chronic gastritis, and peptic ulcers, it is also one of the pathogenic factors for cardiovascular diseases, neurological disorders, hematological diseases, and other conditions (2, 3). Its etiology is complex, and its pathogenesis remains unclear, making prevention and treatment challenging and imposing a significant burden on global public health. In recent years, the potential association between HP infection and metabolic diseases has become a hotspot in interdisciplinary research, with its mechanisms involving multi-dimensional regulation of microbe-host interactions, immune modulation, and metabolic homeostasis (4–8). Investigating the potential related risks of HP infection is of great significance for its prevention and control. HP infection is associated with various factors, primarily involving transmission routes, lifestyle habits (such as poor hygiene), environmental factors, and host conditions (such as age, genetics, and immunity) (9–11). A studies have also found that HP infection may be potentially linked to metabolic diseases such as obesity (BMI ≥28 kg/m²), diabetes, and hypertension (12, 13).

However, the risk associations remain controversial, and there is currently no research exploring the metabolic-related mediating mechanisms and threshold effects of these metabolic diseases on HP infection. The existing literature on the association between *H. pylori* and metabolic factors, particularly BMI, is notably inconsistent, with studies reporting positive, negative, or null findings. This heterogeneity may stem from several factors, including differences in study populations (e.g., age, ethnicity, baseline metabolic health), variations in the diagnostic methods for *H. pylori* status (active vs. past infection), and inadequate adjustment for confounding variables such as socioeconomic status and diet (14, 15).Furthermore, the prevalence of more virulent *H. pylori* strains (e.g., CagA-positive) may differ across regions, potentially modifying the host’s metabolic response (16).

Therefore, we believe that the H. pylori-BMI association is only observable, or is strongest, in populations with a higher baseline prevalence of the pro-inflammatory state (characterized by high leptin and low adiponectin) or subclinical malnutrition (low albumin) that H. pylori is thought to exacerbate (17).

With the changes in lifestyle, the prevalence of metabolic-related diseases has been increasing exponentially (18). It is estimated that currently over one billion people worldwide suffer from metabolic-related diseases (with a prevalence rate of approximately 25%) (19). Understanding the metabolic factors associated with HP infection and their impact on public health is particularly important.

This study employs a retrospective cross-sectional design to investigate the association between HP infection and metabolic indicators such as Body Mass Index (BMI), blood glucose (fasting blood glucose (FBG), glycosylated hemoglobin (HbA1c)), blood pressure (Systolic Blood Pressure (SBP), Diastolic Blood Pressure (DBP)), and blood lipids. It analyzes the mediating role of BMI and HbA1c in the pathway of HP infection, as well as the threshold effect of BMI in its association with HP. The study aims to elucidate the potential regulatory role of metabolic indicators (such as HbA1c, Albumin (Alb), and blood lipids) in the risk of HP infection. To provide new evidence for the mechanistic association between HP infection and metabolism-related diseases, and to offer a theoretical basis for personalized prevention and public health intervention strategies through regression analysis, threshold effect analysis, and mediation analysis.

## 2 Methods

### 2.1 Participants

The study participants were recruited from the Physical Examination Department and Endocrinology Department of Peking University Shenzhen Hospital between January 2021 and December 2022, who underwent the carbon-13 breath test. Exclusion criteria were: history of gastrointestinal surgery or chronic gastrointestinal diseases, including gastrointestinal tumors, peptic ulcers, reflux esophagitis, and cholecystectomy, use of antibiotics or acid-suppressing drugs (including proton pump inhibitors, H2 receptor antagonists, and bismuth preparations) within the past one month; history of major diseases within the past three months, such as myocardial infarction, stroke, heart failure, severe arrhythmias, and malignant tumors; concurrent acute or critical illnesses; chronic kidney disease stage 3 or above, eGFR <60 ml/min. For Chinese adults, the widely accepted and officially recommended criteria are a BMI of 24.0–27.9 kg/m² for overweight and a BMI 28.0 kg/m² for obesity (20). The study enrolled 7,387 participants, who were categorized into an HP-positive group and an HP-negative control group based on the results of the carbon-13 breath test. This was a retrospective study that did not require ethical approval, and all participants signed informed consent forms upon admission, agreeing to share their health information for medical research.

### 2.2 Clinical measures

The basic information of the participants (BMI, SBP, DBP) was recorded by specialized nurses from Peking University Shenzhen Hospital and randomly sampled and checked by at least one researcher. The enrolled participants confirmed for inclusion testing were instructed to avoid strenuous exercise or excessive water intake, smoking, and caffeine consumption 30 minutes prior to the formal measurements. Blood pressure measurements were taken twice, with the average of two measurements recorded. If the difference between the two measurements exceeded 10 mmHg, a third measurement was taken, and the highest of the three values was used. At the same time, Biochemical indicators, including FBG, HbA1c, Uric Acid (UA), Triglyceride (TG), Total Cholesterol (TC), Total Protein (TP), Direct Bilirubin (DB) were measured in participants after an 8–12 hour fast. Venous blood was collected in test tubes and stored at room temperature (≤25 °C), with analysis conducted within 24 hours. Automated analysis was performed using the AU5800 Series Chemistry Analyzers (Beckman Coulter) at the Clinical Laboratory of Peking University Shenzhen Hospital, with random sampling quality control conducted by specialized laboratory personnel.

The diagnosis of *H. pylori* infection was established using the carbon-13 (¹³C) urea breath test, which detects active infection. Diabetes was defined based on a prior physician diagnosis, current use of antidiabetic medication, or a fasting blood glucose (FBG) level ≥ 7.0 mmol/L or HbA1c ≥ 6.5% at the time of the examination.

### 2.3 Statistical analysis

#### 2.3.1 Basic statistical analysis

Continuous variables following a normal distribution are expressed as mean ± standard deviation (SD), while categorical variables are presented as percentages (%). Continuous variables are analyzed using t-tests, and categorical variables are assessed using chi-square tests, A P-value of ≤0.05 is considered statistically significant with all statistical tests being two-tailed. To identify factors independently associated with H. pylori positivity, we performed multivariable logistic regression. The dependent variable was H. pylori infection status. Candidate predictors included demographic variables (age, sex) and metabolic indicators (BMI, SBP, DBP, FBG, HbA1c, UA, TP, Alb, TG, TC, DB) as well as diabetes status. We developed four models with sequential adjustment to assess the stability of these associations: Model 1: Unadjusted model. Model 2: Adjusted for age, sex, SBP, and DBP. Model 3: Further adjusted for Model 2 covariates plus TP, TG, and TC. Model 4: Fully adjusted model, including all covariates from Model 3 plus UA and DB.

#### 2.3.2 Analysis of mediation

To investigate the potential mediating pathways, we performed mediation analysis. We specifically employed a modern non-parametric bootstrapping approach, as this method overcomes the statistical power limitations and strict distributional assumptions inherent in traditional approaches such as the Baron-Kenny causal steps method (21).

This study employed mediation analysis to explore the pathways through which the independent variables (BMI and diabetic status; X) influence the outcome variable (incidence of HP positivity; Y), with Hemoglobin A1c (HbA1c) and a second measurement of BMI as mediators (M). We employed the Bootstrap method to test the significance of the mediating effect. Thousand bootstraps were used in our analysis. The analytical process involved the following steps: first, we established the total effect of X on Y (β_Tot_); second, we regressed M on X to obtain the path coefficient β_1_; third, we regressed Y on both X and M simultaneously to obtain the direct effect of X (β_Dir_) and the effect of M (β_2_). The indirect effect was estimated by the product of the path coefficients (β_1_ × β_2_), and its proportion of the total effect was calculated as (β_1_ × β_2_)/β_Tot_.

#### 2.3.3 Threshold value analysis

Given BMI showed a strong association, to explore the non-linear relationship between BMI and the risk of HP infection, we performed a threshold effect analysis. The threshold effect refers to the phenomenon where the impact of the independent variable (X) on the dependent variable (Y) exhibits significant differences (such as abrupt changes in linear slope or reversal of effect direction) before and after a certain critical point (threshold). We divide the data into two segments based on the threshold and fit separate regression models for each segment, using a piecewise linear regression model to define intervals and identify threshold effects. We used a piecewise logistic regression approach to test for a threshold in the BMI effect. The model allowed for different slopes of BMI below and above an unknown inflection point, which was estimated from the data by optimizing model fit. A likelihood ratio test compared this two-slope model to a single-slope (no threshold) model. Through threshold analysis, we explore whether the mediating BMI exhibits a threshold effect.

### 2.4 Statistical software

All statistical analyses were performed with the use of R (4.3.3). “mediation” package in R 4.2.3. was utilized to perform Mediation analysis assessing the mediating effects.

## 3 Result

### 3.1 Demographic data and baseline analysis

A total of 7,387 participants were enrolled in this study, with certain differences observed in various indicators between the HP-negative group (n = 5,443) and the HP-positive group (n = 1,943). The age distribution was 44.53 ± 10.51 years in the HP-positive group and 43.98 ± 10.27 years in the HP-negative group (t = -2.00, p = 0.045). The BMI in the HP-positive group was significantly higher than that in the HP-negative group (23.88 ± 2.76 vs 23.61 ± 2.79 kg/m2, t = -3.67, P< 0.001). Both SBP and DBP were also higher in the H. pylori-positive group (SBP: 118.79 ± 16.10 vs 117.77 ± 15.68 mmHg, t = –2.45, P = 0.014; DBP: 71.98 ± 11.01 vs 71.38 ± 10.61 mmHg, t = –2.08, P = 0.037). Additionally, the FBG and HbA1c levels were significantly higher in the H. pylori-positive group (FBG: 5.00 ± 1.05 vs 4.94 ± 0.92 mmol/L, t = –2.54, P = 0.011; HbA1c: 5.53 ± 0.66 vs 5.47 ± 0.53%, t = –3.64, P < 0.001). Among the liver function indicators, the Alb level was significantly lower in the H. pylori-positive group (44.41 ± 2.55 vs 44.79 ± 2.53 g/L, t = 5.61, P < 0.001), while the TC was slightly higher (5.26 ± 0.95 vs 5.20 ± 0.97 mmol/L, t = –2.15, P = 0.031). Conversely, there were no significant differences between the two groups in UA, T-BIL, DB, TP, and TG (all P > 0.05). In terms of categorical variables, the gender distribution was similar between the two groups (χ² = 0.06, P = 0.804), but the prevalence of diabetes was significantly higher in the HP-positive group compared to the HP-negative group (4.73% and 3.34%, respectively; χ² = 7.76, P = 0.005). (**Table 1**) It is important to note that while many differences were statistically significant due to the large sample size, the absolute magnitude of some differences was small (e.g., a mean BMI difference of 0.27 kg/m²), suggesting considerable overlap in the distributions between the HP-positive and HP-negative groups.

**Table 1:** Analysis of basic characteristics and differences.

Variables	Total (n = 7386)	Negative (n = 5443)	Positive (n = 1943)	Statistic	*P*
Age (years), Mean ± SD	44.12 ± 10.33	43.98 ± 10.27	44.53 ± 10.51	t=-2.00	**0.045**
BMI (kg/m2), Mean ± SD	23.68 ± 2.79	23.61 ± 2.79	23.88 ± 2.76	t=-3.67	**<.001**
SBP (mmHg), Mean ± SD	118.03 ± 15.80	117.77 ± 15.68	118.79 ± 16.10	t=-2.45	**0.014**
DBP (mmHg), Mean ± SD	71.53 ± 10.72	71.38 ± 10.61	71.98 ± 11.01	t=-2.08	**0.037**
FPG (mmol/L), Mean ± SD	4.95 ± 0.96	4.94 ± 0.92	5.00 ± 1.05	t=-2.54	**0.011**
HbA1c (%), Mean ± SD	5.49 ± 0.57	5.47 ± 0.53	5.53 ± 0.66	t=-3.64	**<.001**
UA (μmol/L), Mean ± SD	360.28 ± 91.73	360.10 ± 91.16	360.77 ± 93.35	t=-0.27	0.784
TBIL (μmol/L), Mean ± SD	15.18 ± 5.84	15.24 ± 5.88	15.00 ± 5.72	t=1.55	0.120
DB (μmol/L), Mean ± SD	2.80 ± 1.10	2.81 ± 1.14	2.76 ± 0.99	t=1.73	0.084
TP(g/L), Mean ± SD	75.72 ± 4.07	75.74 ± 4.05	75.65 ± 4.13	t=0.85	0.396
Alb(g/L), Mean ± SD	44.69 ± 2.55	44.79 ± 2.53	44.41 ± 2.55	t=5.61	**<.001**
TG (mmol/L), Mean ± SD	1.48 ± 0.93	1.48 ± 0.94	1.48 ± 0.89	t=-0.05	0.964
TC (mmol/L), Mean ± SD	5.22 ± 0.97	5.20 ± 0.97	5.26 ± 0.95	t=-2.15	**0.031**
Sex, n (%)				χ²=0.06	0.804
male	4666 (63.17)	3434 (63.09)	1232 (63.41)		
female	2720 (36.83)	2009 (36.91)	711 (36.59)		
Diabetes, n (%)				χ²=7.76	**0.005**
negative	7112 (96.29)	5261 (96.66)	1851 (95.27)		
positive	274 (3.71)	182 (3.34)	92 (4.73)		

Data were presented as mean ± standard deviation or number (%) as appropriate.

A total of 7386 participants were included in this study, of which 5443 participants had H.pylori negative, accounting for 73.69%, and 1943 participants had H.pylori positive, accounting for 26.31%. There were significant differences in BMI, SBP, DBP, FPG, HbA1c, Alb, TC, and Diabetes between the two groups (P<0.05), but no significant differences in UA, TBIL, DB, TP, TG, and Sex (P>0.05).

The bolded font indicates P < 0.05.

### 3.2 Binary logistic regression analysis

In the multivariate logistic regression analysis, Age, BMI, SBP, DBP, FBG, HbA1c, Alb, TC, and Diabetes were all significantly associated with the target outcome (P<0.05). Among these, for each 1-year increase in age, there was a slight elevation in the risk of the outcome ((OR = 1.04, 95% Confidence Interval (CI): 1.02–1.06, P<0.001)); both SBP and DBP also showed a slight but statistically significant positive association (OR = 1.01 for both, P<0.05). Among the glycemic metabolism indicators, FPG and HbA1c have particularly prominent effects on outcome risk, with odds ratio (OR) of 1.07 (95% CI: 1.02–1.13, P = 0.007) and 1.19 (95% CI: 1.09–1.29, P<0.001), respectively. In contrast, Alb showed a negative correlation with outcome risk (OR = 0.94, 95% CI: 0.92–0.96, P<0.001), suggesting a potential protective effect. Among the lipid-related indicators, TC reached statistical significance (OR = 1.06, 95% CI: 1.01–1.12, P = 0.031), whereas TG showed no significant association with outcome risk (P = 0.964). It is noteworthy that, compared to non-diabetic individuals, those with diabetes exhibited a significantly elevated risk of adverse outcomes (OR = 1.44, 95% CI: 1.11–1.86, P = 0.006). Additionally, UA, T-BIL, DB, TP, and Sex did not demonstrate significant influence in this model (P>0.05). Overall, our analysis identified that factors such as blood glucose levels (FBG, HbA1c), BMI, and blood pressure (SBP, DBP) were strongly and independently associated with the risk of HP infection. (**Table 2**).

**Table 2:** Univariate logistic regression results.

Variables	β	S.E	Z	*P*	OR (95%CI)
Age (years)	0.01	0.00	2.00	**0.045**	1.01 (1.01 ~ 1.01)
BMI	0.03	0.01	3.67	**<.001**	1.04 (1.02 ~ 1.06)
SBP	0.01	0.00	2.44	**0.015**	1.01 (1.01 - 1.01)
DBP	0.01	0.00	2.12	**0.034**	1.01 (1.01 ~ 1.01)
FPG	0.07	0.03	2.68	**0.007**	1.07 (1.02 ~ 1.13)
HbA1c	0.17	0.04	3.95	**<.001**	1.19 (1.09 ~ 1.29)
UA	0.00	0.00	0.27	0.784	1.00 (1.00 ~ 1.00)
TBIL	-0.01	0.00	-1.55	0.120	0.99 (0.98 ~ 1.00)
DB	-0.04	0.03	-1.73	0.083	0.96 (0.91 ~ 1.01)
TP	-0.01	0.01	-0.85	0.396	0.99 (0.98 ~ 1.01)
ALB	-0.06	0.01	-5.59	**<.001**	0.94 (0.92 ~ 0.96)
TG	0.00	0.03	0.05	0.964	1.00 (0.95 ~ 1.06)
TC	0.06	0.03	2.15	**0.031**	1.06 (1.01 ~ 1.12)
Sex					
male					1.00 (Reference)
female	-0.01	0.05	-0.25	0.804	0.99 (0.89 ~ 1.10)
Diabetes					
negative positive	0.362	0.131	2.772	0.006	1.000 (Reference) 1.437 (1.112 ~ 1.856)

OR, Odds Ratio; CI, Confidence Interval.

The bolded font indicates P < 0.05.

### 3.3 Logistics multiple model regression

Logistic regression analysis on the risk of HP infection revealed that in the unadjusted model (Model 1), HbA1c, BMI, FBG, and diabetes were all significantly positively associated with the target outcome (P<0.05), while Alb exhibited a significant negative correlation (OR = 0.94, 95% Confidence Interval (CI): 0.92–0.96, P<0.001). In the models that progressively incorporated confounding factors (Model 2 to Model 4), the associations between these variables and the target outcome generally remained stable or slightly attenuated, but most still retained statistical significance (P<0.05), suggesting their independent effects. In the fully adjusted model (Model 4) (adjusted for Sex, Age, SBP, DBP, UA, DB, TP, TG, TC), each 1-unit increase in HbA1c was associated with an approximately 17% higher risk of the outcome (OR = 1.17, 95% CI: 1.07–1.28, P = 0.001); each 1-unit increase in BMI was associated with an approximately 4% higher risk (OR = 1.04, 95% CI: 1.02–1.06, P<0.001); FBG also showed a positive association (OR = 1.06, 95% CI: 1.01–1.12, P = 0.030); while each 1-unit increase in Alb was associated with an approximately 8% lower risk of the outcome (OR = 0.92, 95% CI: 0.90–0.94, P<0.001). Additionally, the risk for individuals with diabetes increased by approximately 40% (OR = 1.40, 95% CI: 1.07–1.82, P = 0.013). The above results indicate that glycemic indicators, BMI, and diabetes status significantly promote the target outcome, while higher levels of Alb may exert a certain protective effect. (**Table 3**).

**Table 3:** Multiple logistic regression model.

Variables	Model1		Model2		Model3		Model4	
	OR (95%CI)	*P*	OR (95%CI)	*P*	OR (95%CI)	*P*	OR (95%CI)	*P*
HbA1c	1.19 (1.09 ~ 1.29)	**<.001**	1.17 (1.07 ~ 1.28)	**<.001**	1.17 (1.07 ~ 1.28)	**<.001**	1.17 (1.07 ~ 1.28)	**0.001**
BMI	1.04 (1.02 ~ 1.06)	**<.001**	1.04 (1.01 ~ 1.06)	**0.001**	1.04 (1.02 ~ 1.06)	**<.001**	1.04 (1.02 ~ 1.06)	**<.001**
FPG	1.07 (1.02 ~ 1.13)	**0.007**	1.06 (1.01 ~ 1.11)	**0.045**	1.06 (1.01 ~ 1.12)	**0.034**	1.06 (1.01 ~ 1.12)	**0.030**
ALB	0.94 (0.92 ~ 0.96)	**<.001**	0.94 (0.91 ~ 0.96)	**<.001**	0.92 (0.89 ~ 0.94)	**<.001**	0.92 (0.90 ~ 0.94)	**<.001**
Diabetes	1.44 (1.11 ~ 1.86)	**0.006**	1.36 (1.04 ~ 1.77)	**0.022**	1.38 (1.06 ~ 1.80)	**0.017**	1.40 (1.07 ~ 1.82)	**0.013**

OR, Odds Ratio; CI, Confidence Interval;

Model1: Crude.

Model2: Adjust: Sex, Age (years), SBP, DBP.

Model3: Adjust: Sex, Age (years), SBP, DBP, TP, TG, TC.

Model4: Adjust: Sex, Age (years), SBP, DBP, UA, DB, TP, TG, TC.

The bolded font indicates P < 0.05.

### 3.4 Analysis of mediation

**Figure 1:**
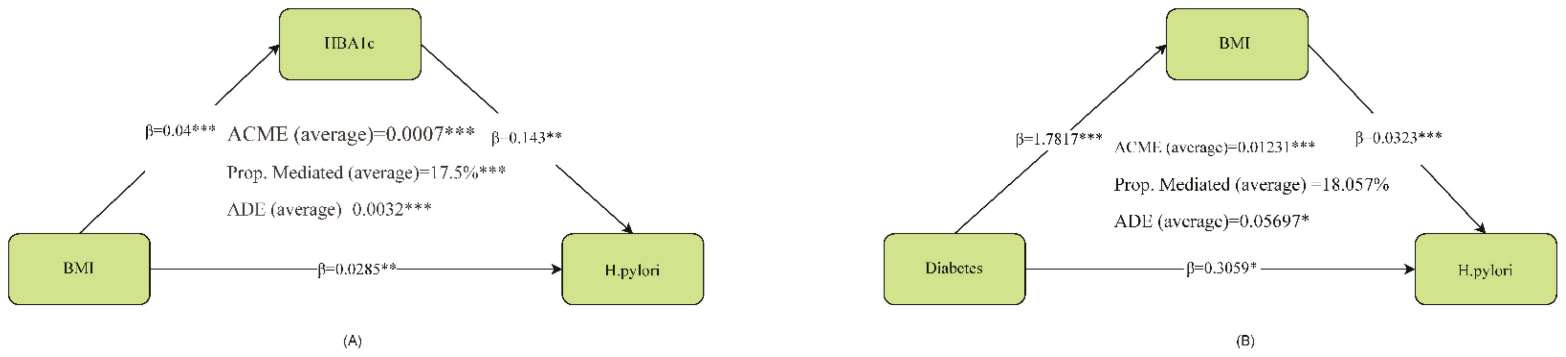
Analysis of mediation. ACME, average causal mediation effect; ADE, average direct effect *represents p<0.05, **represents p<0.01, ***represents p<0.001. HBA1c partially mediated the relationship between BMI and H. pylori. In conclusion, BMI partially mediated the relationship between Diabetes and H. pylori.

The figure below illustrates the results of the mediation analysis with HBA1c as the mediating variable and BMI’s impact on HP. It is necessary to clarify that the directionality (e.g., BMI → HbA1c → H. pylori) is assumed based on known biology, not directly proven by the data. Firstly, the effect of BMI on HBA1c is significant (β = 0.04, p < 0.001), and the effect of HBA1c on H. pylori is also significant (β = 0.143, p < 0.01). Further analysis of the mediation effect reveals that the average causal mediation effect (ACME) of BMI on HP through HBA1c is 0.0007 (p < 0.001), accounting for 17.5% of the total effect (p < 0.001). Meanwhile, the average direct effect (ADE) of BMI on HP was 0.0032 (p < 0.001). Additionally, the direct path coefficient of BMI on HP also reached a significant level (β = 0.0285, p < 0.01). In summary, HBA1c played a partial mediating role between BMI and HP. In the mediation analysis with BMI as the mediating variable and the effect of Diabetes on HP, the impact of Diabetes on BMI was significant (β = 1.7817, p < 0.001), and the effect of BMI on HP was also significant (β = 0.0323, p < 0.001). Further mediation effect analysis revealed that the average causal mediation effect (ACME) of Diabetes on HP through BMI was 0.01231 (p < 0.001), accounting for 18.057% of the total effect, with p < 0.04. Simultaneously, the average direct effect (ADE) of Diabetes on HP was 0.05697 (p < 0.05). Additionally, the direct path coefficient of Diabetes on HP also reached a significant level (β = 0.3059, p < 0.05). In summary, BMI partially mediated the relationship between Diabetes and HP. **(.Analysis of mediation****Figure 1**).

### 3.5 Threshold value analysis

**Figure 2:**
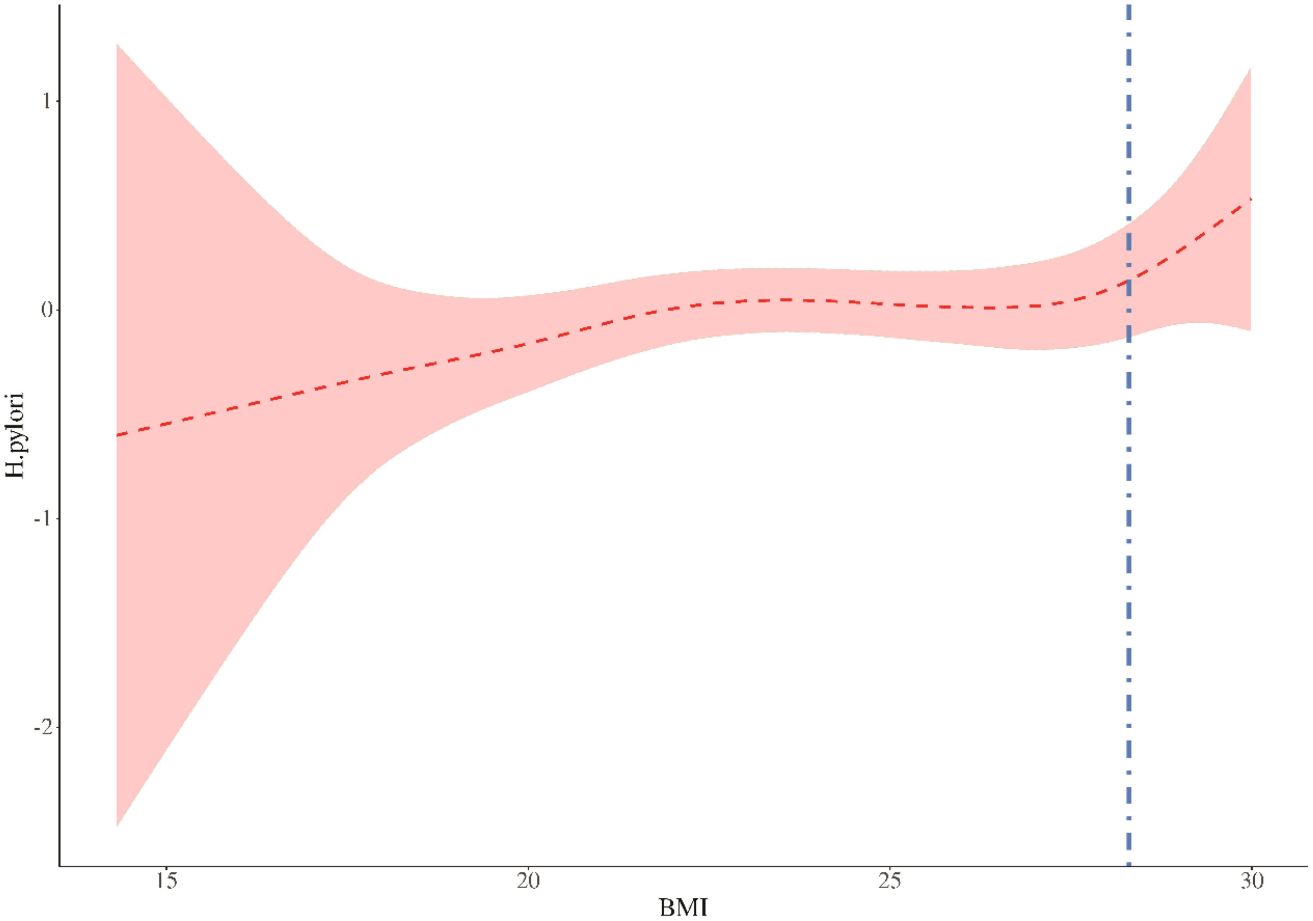
Threshold value analysis. In two-stage regression, the inflection point was 28.298. When expose BMI 28.298, P=0.005. The vertical dashed line indicates the critical value of 28.298.

Threshold analysis revealed a threshold effect in the association between BMI and HP (P for likelihood test = 0.030). Overall, BMI and HP were positively associated [OR (95% CI): 1.04 (1.02 - 1.06)]. When BMI was below 28.298kg/m^2^, BMI and HP were positively associated [OR (95% CI): 1.03 (1.01 - 1.05)]. When BMI was above 28.298 kg/m^2^, BMI and HP were positively associated [OR (95% CI): 1.91 (1.22 - 3.00)], with an 88% increased risk, demonstrating a significant threshold effect. (**Table 4**, **Figure 2**).

**Table 4:** Threshold value analysis.

Outcome	effect	*P*
Model 1 Fitting model by standard linear regression	1.04 (1.02 - 1.06)	**<.001**
Model 2 Fitting model by two-piecewise linear regression		
Inflection point	28.298	
<28.298	1.03 (1.01 - 1.05)	**0.011**
≥28.298	1.91 (1.22 - 3.00)	**0.005**
P for likelihood test		**0.030**

There was a threshold effect for the association between BMI and H.pylori (P for likelihood test = 0.030). In general, the association between BMI and H.pylori was positive [OR (95%CI): 1.04 (1.02-1.06)]. When BMI was lower than 28.298, BMI was positively associated with H.pylori [OR (95%CI): 1.03 (1.01-1.05)]. When BMI was higher than 28.298, BMI was positively associated with H.pylori [OR (95%CI): 1.91 (1.22-3.00)].

The bolded font indicates P < 0.05.

## 4 Discussion

The detection of HP can be conducted through several different invasive or non-invasive methods, each with its own advantages and disadvantages. The urea breath test is the most commonly used non-invasive diagnostic method due to its relatively low cost, high sensitivity, and specificity (22). However, few patients actively screen for HP infection during routine check-ups. HP infection may lead to severe diseases of the digestive system, including gastritis and peptic ulcers, gastric cancer, and gastric mucosa-associated lymphoid tissue lymphoma (23–25). HP infection increases the risk of metabolic syndrome by approximately 15%. Long-term gastric inflammation may disrupt metabolic balance through mechanisms such as influencing gastric hormones.^8^ Identifying high-risk factors related to HP infection is extremely important for some potentially high-risk populations.

In our study, it was found that the H. pylori-positive group had significantly higher BMI, blood pressure, blood glucose indicators (FPG, HbA1c), and prevalence of diabetes compared to the negative group, with decreased Alb levels, suggesting that Alb may be a protective factor. We further conducted multivariate logistic regression to validate that BMI (OR = 1.04, P<.001), HbA1c (OR = 1.19, P<0.001), FPG (OR = 1.07, P<0.05), and diabetes (OR = 1.44, P<0.01) are significant risk factors for H. pylori infection, while Alb (OR = 0.94, P<0.001) exhibits a protective effect. Subsequently, we adjusted relevant variables to explore the independent contribution of each variable to the event risk. We found that after three rounds of adjustments, BMI, HbA1c, FPG, and diabetes still demonstrated strong associations, and Alb continued to show a protective effect against H. pylori infection. This provides stronger evidence for our previous findings and indicates their strong independent relevance to H. pylori infection. According to our results, BMI, HbA1c, FPG, and diabetes are risk factors for HP infection. It can be inferred that diabetes has a significant association with HP infection, which further validates the conclusion from multiple meta-analyses indicating that the HP infection rate is higher in diabetic patients than in non-diabetic populations (26, 27). There may be several reasons for this. Diabetic patients are often prone to abnormal glucose metabolism, which can lead to immune damage in the body, weakening the chemotaxis, phagocytosis, and bactericidal abilities of immune cells such as neutrophils and macrophages (28–30). This may make patients more susceptible to Hp infection. Secondly, from the perspective of gastrointestinal metabolism, diabetes may cause gastrointestinal motility disorders, reduced gastric acid secretion, and microangiopathy, which decreases gastric mucosal blood flow and delays the repair of damaged mucosa, thereby promoting the colonization of H. pylori (31). Currently, there is limited research exploring the relationship between plasma Alb and HP infection. Some studies have shown a higher prevalence of HP infection in patients with hypoalbuminemia, such as those with cirrhosis or nephrotic syndrome, suggesting a potential negative correlation between Alb levels and HP susceptibility (32, 33). Based on our findings, Alb appears to have a protective effect. As an important antioxidant and anti-inflammatory protein in plasma, Alb that exudes during gastric mucosal damage (such as inflammation or ulcers) can scavenge free radicals, mitigate oxidative damage caused by HP infection, and delay mucosal destruction (34). Furthermore, Alb may competitively bind to adhesins on the surface of HP (such as the BabA protein), reducing its attachment to gastric epithelial cells (35). While our finding of a protective association for albumin aligns with literature suggesting that malnutrition or systemic inflammation (for which low albumin is a marker) can increase infection susceptibility, contradictory evidence exists. Notably, a study by **Jalalzadeh et al. (**36) In hemodialysis patients found that successful H. pylori eradication led to a decrease in serum albumin. This discrepancy may be attributable to the profound differences in the underlying metabolic and inflammatory states between the general population and patients with end-stage renal disease, where the drivers of albumin concentration are highly complex.

Interestingly, while glycemic markers and BMI were strongly associated with *H. pylori*, serum triglycerides (TG) showed no association. This suggests that the link between metabolic dysregulation and *H. pylori* may be more specifically related to pathways of adiposity and glucose metabolism rather than dyslipidemia in general (37).

While our study did not measure all inflammatory markers, our findings, when integrated, suggest that *H. pylori* infection is associated with a broader phenotype of low-grade, obesity-driven inflammation. The strong association with high BMI and impaired glycemic control (HbA1c) points towards a state of metabolic stress. This condition is known from extensive literature to be characterized by an imbalance in key adipokines—specifically, elevated levels of the pro-inflammatory hormone leptin (often with leptin resistance) and reduced levels of the anti-inflammatory hormone adiponectin (38). Although these adipokines were not measured in our cohort, the observed lower levels of serum albumin are consistent with the presence of a chronic systemic inflammatory state.

Therefore, we hypothesize that the link between BMI and *H. pylori* is not merely due to weight itself, but to the associated pro-inflammatory endocrine environment created by dysfunctional adipose tissue. Future prospective studies should concurrently measure leptin and adiponectin to test this hypothesis directly, perhaps by using the Leptin-to-Adiponectin Ratio (LAR) as a more robust composite mediator. Such an approach would provide a more complete biological narrative and further clarify the mechanisms underlying the increased susceptibility to *H. pylori* in individuals with obesity.

To further explore other possible pathogenic mechanisms, we conducted mediation and threshold analyses. In the large-scale systematic review and meta-analysis conducted by Baradaran A et al., it was found that obese individuals had a significantly higher risk of contracting Helicobacter pylori, but no discussion was made regarding the mediating effect or threshold. In our mediation analysis, we found that the relationship between BMI and H. pylori was partially mediated by HbA1c, with an estimated 17.5% of the total effect occurring through this indirect pathway. Furthermore, our threshold analysis identified a BMI inflection point of 28.298 kg/m²; above this threshold, the risk of H. pylori infection increased by 88%. This suggests that a portion of the association between higher BMI and HP infection risk may be explained by elevated glucose levels as measured by HbA1c. This finding demonstrates a strong positive association, where the likelihood of detecting H. pylori infection is significantly higher in individuals with a high BMI (39). There is a correlation between HP seropositivity and body weight, waist circumference, and BMI (40, 41). The risk of HP infection is positively correlated with the prevalence of obesity, and our findings corroborate this point. This suggests that regular follow-up and re-examination for HP infection are particularly important for this population. The intestinal immune function of obese individuals is impaired, and the ability of monocytes to transform into macrophages is reduced in these patients (42, 43). In obese individuals, the cytotoxic activity of natural killer cells is normal compared to healthy individuals with a normal BMI, making them more susceptible to HP infection (44, 45). In morbidly obese patients, the ability of monocytes to transform into macrophages is reduced, and preadipocytes can exhibit phagocytic activity against microorganisms such as macrophage-like cells until they cease proliferation and differentiate into adipocytes (46, 47). These potential mechanisms well explain why the immune environment in excessively obese patients is more conducive to the colonization of HP.

These research results have significant clinical and public health implications. For clinicians, they indicate that for patients with symptoms of digestive disorders and a high body mass index (BMI) (particularly above 28 kilograms per square meter, which is roughly the dividing line between overweight and obesity in the Asian population), there should be a lower threshold for conducting Helicobacter pylori testing. From a public health perspective, our research results further emphasize the importance of controlling the prevalence of obesity and diabetes, as improving metabolic health may be a strategy to reduce susceptibility to chronic infections such as Helicobacter pylori. For some resource-poor areas, conducting HP testing for obese or diabetic patients can help increase the detection rate of infections. At the same time, for regions with high prevalence of Helicobacter pylori and metabolic syndrome, comprehensive treatment may be beneficial for the comprehensive management of the disease, which may break a vicious cycle, as some evidence suggests that Helicobacter pylori itself may cause metabolic disorders.

## 5 Conclusion

In conclusion, this large-scale cross-sectional study demonstrated that the key components of metabolic syndrome, particularly high body mass index and poor glycemic control (elevated fasting blood glucose and elevated glycated hemoglobin), were significantly and independently positively correlated with the risk of Helicobacter pylori infection in adults. Higher albumin levels seemed to have a protective effect. Our analysis provided new mechanistic insights, indicating that the link between body mass index and Helicobacter pylori infection is partially mediated by the glycemic state. Importantly, we identified a body mass index threshold of approximately 28.3 kg/m², beyond which the risk of infection increases sharply.

## 6 Limitations

Due to the cross-sectional nature of our study, we were unable to establish causality but could only explore associations. Additionally, the retrospective design limited our ability to control for potential biases introduced by family history, which should be further addressed in future research designs. The study population primarily consisted of hospital visitors or individuals undergoing health check-ups, potentially introducing a “healthcare-seeking bias.” Further follow-up investigations are needed for asymptomatic individuals or those who did not seek medical attention. Future studies should employ prospective cohort designs, multi-center samples, more comprehensive control of confounding factors, and exploration of experimental mechanisms to further validate and deepen the current conclusions. This study effectively explored the potential risk and protective factors for HP infection based on real-world cases. We were the first to reveal the threshold effect of BMI on HP infection, but whether this threshold has universal clinical significance still requires validation through prospective studies. Meanwhile, our research focused on statistical associations, yet it did not provide a deeper understanding of the underlying biological mechanisms (such as the protective effect of Alb and the obesity-related immunosuppressive mechanisms). Future studies could focus on experimental methods to validate hypotheses.

## Data Availability

The relevant data in this paper involve the privacy data of hospitals and patients. Please contact the corresponding author for relevant data needs. Requests to access the datasets should be directed to X-fL, lxf198804@163.com.
